# Data on the dynamics of landscape structure and fragmentation in Ambo district, central highlands of Ethiopia

**DOI:** 10.1016/j.dib.2021.106782

**Published:** 2021-01-21

**Authors:** Fanta Obsa, Berhanu Kefale, Moges Kidane, Terefe Tolessa

**Affiliations:** a Institute of Land Administration, Bahirdar University, Ethiopia; b College of Agriculture and Veterinary Sciences, Ambo University, Ethiopia; c Institute of Cooperatives and Development Studies, Ambo University, Ethiopia

**Keywords:** Land use/Land cover, Fragmentation, Landscape, Metrics, FRAGSTAT

## Abstract

The data presented in this article show changes in land use/land cover and fragmentation of land at a landscape level for a period of 45 years (1973-2018) in Ambo district of the central highlands of Ethiopia. Data generated from satellite images of Multispectral Scanner (MSS), Enhanced Thematic Mapper (ETM) and Operational Land Image (OLI) with path/raw value of 181/54, 169/54 and169/54 for each images respectively were analyzed by Arc GIS 10.1 software using a standard method. The precision of the images were verified by data collected from ground control points by using Geographic Positioning System (GPS) receiver. A raster data of LULC was used as an input in to FRAGSTAT software to analyze fragmentation at the landscape level. The data presented in this article showed that cultivated land and settlement increased by 45.7% (376.5ha/yr) and 111% (78.3ha/yr) for 1973-2018 periods respectively. Forest land, shrub land and bare land shrunk by 38% (147.5ha/yr), 17.1% (88.5ha/yr) and 63.9% (218ha/yr) respectively over the periods considered. Transition matrix indicated that 64781.86ha of land unchanged over the years (1973-2018). Number of patches increased by 143% while largest patch index increased by 226% in the years (1973-2018). In contrast, however, Aggregation index has shown a negative value (9.3%) and other metrics such as SIDI (12) and IJI (8.1) has shown an overall decreasing trend.

## Specifications Table

**Table:** 

Subject	Landscape study, Geography
Specific subject area	Land use/Land cover change, Fragmentation
Type of data	Table Figure Text file
How data were acquired	Data were extracted from MSS, ETM and OLI images with path/row values 181/54, 169/54 and169/54 for each images respectively and were analyzed by Arc GIS 10.1 software using a standard method. The precision of the images were verified by data collected from ground control points by using Geographic Positioning System (GPS). After data on LULC change was obtained, a raster data of LULC was as an input in to FRAGSTAT software to analyze fragmentation.
Data format	Raw data
Parameters for data collection	Satellite images were used for data collection and selected cloud free date to improve data quality. The date of acquisition of data also focused on major policy and government change in Ethiopia
Description of data collection	Satellite images selected were downloaded from USGS website which is freely available and Output of FRAGSTAT software
Data source location	City/Town/Region: Ambo district Country: Ethiopia Latitude and longitude: 8o 59′1" N latitude and 37o 51′ E https://earthexplorer.usgs.gov
Data accessibility	http://dx.doi.org/10.17632/mx8bsw5fr7.1

## Value of the Data

• The data is important for the study area to show the level of land use changes and fragmentation so that it can be used to propose appropriate land management practices.
• The data provides evidence on the trends of land use dynamics and level of fragmentation to Ambo district land use planners and the wider scientific community.
• The data can also be useful to researchers, rural land use planners and experts working in the field.

## 1 Data Description

**Fig. 1:**
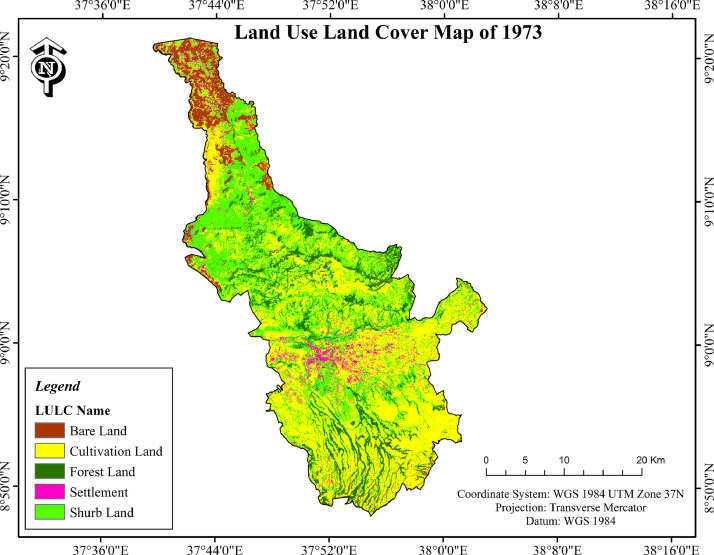
LULC change of Ambo district in 1973.

**Fig. 2:**
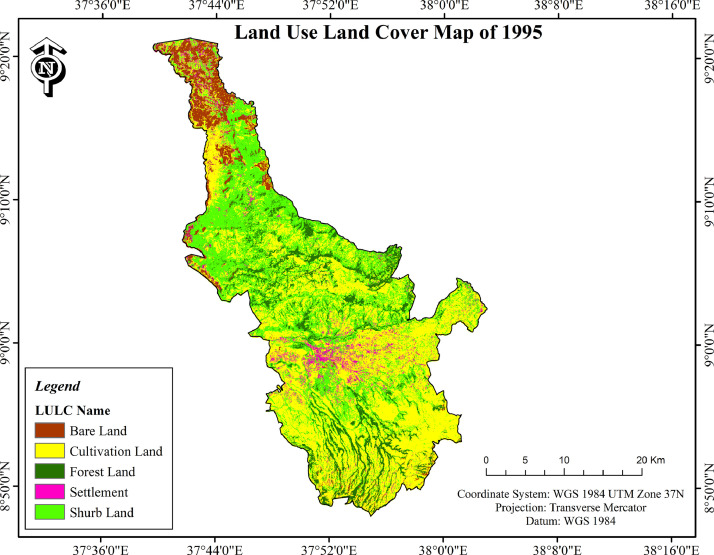
LULC change of Ambo district in 1995.

**Fig. 3:**
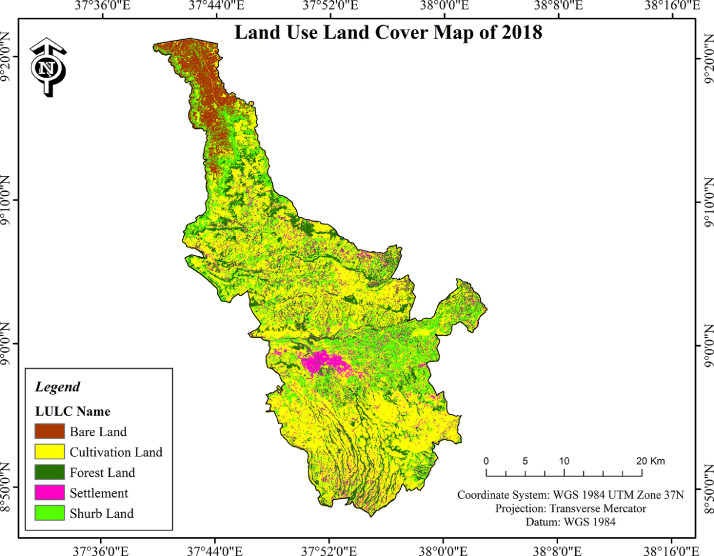
LULC change of Ambo district in 2018.
• Cultivated land and settlement consistently increased within the three study periods but the increase is not uniform (Table 1; Figs. 1–3). In terms of the area coverage cultivated land increased by 32.9%, 9.6% and 45.7% for the periods of 1973-1995, 1995-2018 and 1973-2018 respectively. At the same time settlement increased by 38.4%, 52.5% and 111% for the same periods as indicated on Table 1.

The data described in this data paper are of three different types related to landscape changes and fragmentation for the period of forty five years (1973 – 2018). These are:

• Raw satellite image (.tif) of Ambo district was downloaded from USGS website which is freely available and the image was obtained when there was cloud free season (Raw Satellite Image Folder as supplementary data). Three maps were produced after it was processed in ArcGIS software (Fig. 1, Fig. 2, Fig. 3). Each spatial layer consists of the land cover classes: Settlement, Cultivated land, Bare land, Forest land and Shrub land.

**Table 1:** Land use/land cover changes of Ambo district (1973–2018).

	Absolute area coverage (ha)			Cover change between periods (%)			Rate of change (ha)		
LULC classes	1973	1995	2018	1973-1995	1995-2018	1973-2018	1973-1995	1995-2018	1973-2018
Cultivated land	37099.41	49330.53	54042.30	32.9	9.6	45.7	+555.96	+204.9	+376.5
Settlement	3173.32	4391.73	6697.53	38.4	52.5	111	+55.4	+100.3	+78.3
Forest land	17426.30	15379.26	10790.20	−11.7	−29.8	−38	−93	−199.5	−147.5
Shrub land	23210.68	16827.30	19230.40	−27.5	14.3	−17.1	−290.2	+104.5	−88.5
Bare land	15405.70	10386.59	5554.98	−32.6	−46.5	−63.9	−228.1	−209.9	−218.9
Total	**96315.41**	**96315.41**	**96315.41**
• In contrast to cultivated land and settlement, forest land and bare land shrunk over the study periods (Table 1; Plante1: B) whereas shrub land swung between the two ends. When the dynamics of different land uses are compared within the study period considered cultivated land is highly eroded by gully erosion (Plate 1: A). Bare land serve as source of quarry for construction purposes (Plate 1: C). Mosaics of land uses in the human modified landscape in the study area are dominated by cultivated land (Plate 1: D & E).

**Plate 1:**
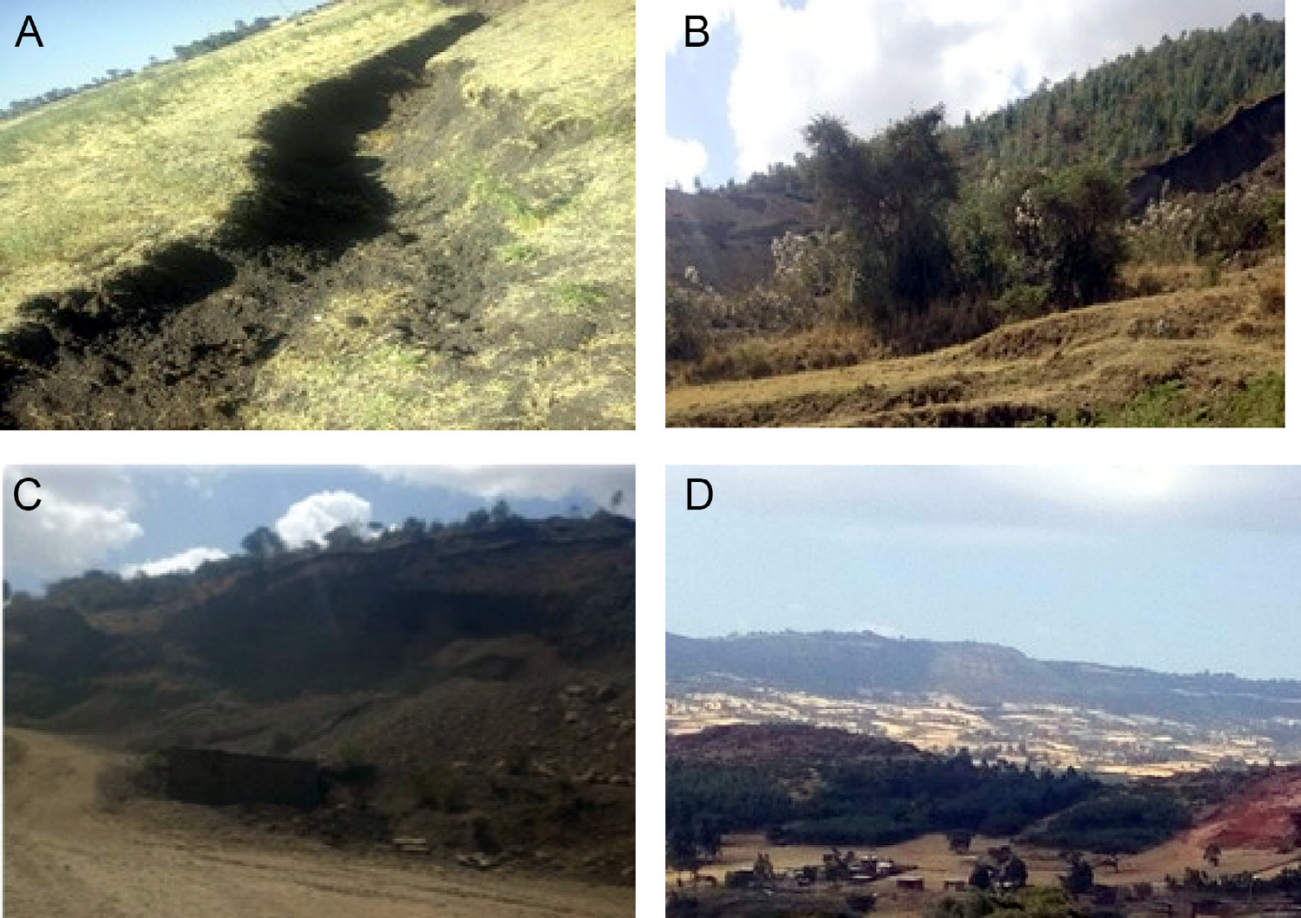
View of some of the LULC classes in Ambo district (A: cultivated land affected by gully erosion); (B: Forest land edge after the forest was removed at the edge on sloppy areas); (C: Bare land used as quarry for construction purposes); (D & E: Mosaics of land uses in the human modified landscape in the study area).
• The highest loss recorded for the study period (1973-2018) was in the shrub land use type (13238.23ha) while the lowest was for settlement (6.12ha). On the other hand, the highest gain was documented for cultivated land (17197.15ha) and lowest gain was for bare land (632.37ha). Overall, 64781.86ha of the total study area was unchanged from which cultivated land dominated the total amount of land use unchanged to be at 56.9% (Table 3). The net change‐to‐persistence ratio was negative for bare land, positive for settlement, negative for forest land, positive for cultivated land, and negative for Shrub land (Table 2).

**Table 2:** Land use/land cover transition matrix of the major changes in Ambo district (1973–2018).

	To final state (2018)						
	Cultivated land	Settlement	Forest land	Shrub land	Bare land	Total 1973	Loss
**From initial state**							
**Cultivated land (ha)**	**36844.95**	9	0.67	9.29	235.5	37099.41	254.46
**Settlement (ha)**	1.76	**3167.2**	0.63	3.39	0.34	3137.32	6.12
**Forest land (ha)**	2096.07	683.9	**9874.33**	4659.59	113.67	17426.3	7553.23
**Shrub land (ha)**	10967.32	1074.99	912.94	**9972.57**	282.86	23210.68	13238.11
**Bare land (ha)**	4132.0	1762.44	2.89	4585.56	**4922.81**	15405.7	10482.89
						a**64781.86**	
**Total 2018**	54042.3	6697.53	10790.2	19230.4	5554.98		
Gain(ha)	17197.15	3530.33	917.13	9257.83	632.37		
Net change^b^(ha)	16942.69	3524.21	−6636.1	−3980.28	−9850.52		
Net persistence (NP)^c^	0.46	1.11	−0.67	−0.40	−2.0		

Bolded diagonal elements represent proportions of each land use/land cover class that were static (persisted) between 1973 and 2018. The loss column and gain row indicate the proportion of the landscape that experienced gross loss and gain in each class, respectively.

All the figures in the table are in hectare except Np, which is a ratio which is the ratio of net change-to-persistence ratio.

a The shaded figure is the sum of diagonals and represents the overall persistence (i.e., the landscape that did not change).

b Net change = gain–loss.

c Np refers to net change‐to‐persistence ratio (i.e., net change/diagonals of each class).
• Raster data sets (.tif) generated for LULC analysis were used as an input for analysis of landscape fragmentation (Raster dataset folder as supplementary data). Number of patches and largest patch index increased by 143% and 226% respectively for the study period (1973-2018) considered. In contrast, however, Aggregation index has shown a negative value (9.3%) and other metrics such as SIDI (12) and IJI (8.1) has shown a decreasing trend (Table 2). Simpson's diversity index (SIDI) decreased from 1973 to 1995, but increased slightly for 1995 -2018 period. The interspersion and juxtaposition index (IJI) decreased for 1971 - 1995 (Table 3; Fragmentation Metrics Folder as supplementary data).

**Table 3:** Landscape metrics for Ambo district (1973–2018).

	Study years			Change between periods %)		
Type of metrics	1973	1995	2018	1973-1995	1995-2018	1973-2018
NP	27432	37040	66585	35	79.8	143
LPI	6.36	20.96	20.76	229	−0.95	226
AI	79.39	77.46	72.02	−2.4	−7	−9.3
LSI	108.25	118.08	146.05	9.1	23.7	34.9
SIDI	0.75	0.65	0.66	−13.3	1.5	−12
IJI	85.67	65.46	78.73	−23.6	20.3	−8.1

## 2 Experimental Design, Materials and Methods

### 2.1 Land use/Land cover data analysis

Three satellite images for three years (1973, 1995 and 2018) with 30 * 30 m spatial resolution were used for LULC change analysis. Geometric, radiometric and atmospheric corrections were conducted using the standard procedures [1]. The satellite images obtained for the study period were geo-referenced corresponding to the Clarke 1880 spheroid and the Adindan UTM (Universal Transverse Mercator) projections. Both Supervised and unsupervised classification technique with maximum likelihood classification methods were used to classify LULC [2], [3], [4]. Different accuracy assessment methods were used for determining the precision of the classification. Various measures of accuracy assessment such as overall accuracy and Kappa coefficient were done [5], [6]. The overall classification accuracy and kappa statistics were 88% and 0.81 respectively. Furthermore, in order to calculate the percentage change in LULC changes between periods, we employed the following equation:

(1) $$\Delta A=\frac{A_{t2}-A_{t1}}{A_{t1}}\times 100$$

where, ΔA(%) refers to the change in the percentage of land use between periods, $A_{t2}$ is the area of the land use at the year t2 and $A_{t1}$ is the area of the land use at year t1.

We have also calculated the rate of change of LULC changes between years to estimate the spatial changes in land uses with the equation:

(2) $$\Delta R=\frac{Y-x}{t}$$

where ΔR(ha/yr) is the rate of change in land use per year, Y is the recent area of the land use and x is the area of the previous land use and t refers to the time interval between the study periods considered for analysis.

Computing landscape fragmentation metrics

In order to compute landscape fragmentation metrics a raster data generated by the use of ArcGIS software was used as an input in the FRAGSTATS software package [7]. Many landscape metrics can be used to analyze spatial patterns of landscape but in this study, ecologically important landscape metrics were selected based on the aim of this study. The metrics include Number of Patches (NP), Largest patch index (LPI), Aggregation index (AI), Simpson's diversity index (SIDI), Interspersion and juxtaposition index (IJI) and Landscape shape index (LSI) (Table 4).

**Table 4:** List of computed Landscape metrics.

Metric	Description
Number of patches (NP)	Total number of patches in the landscape
Largest Patch Index (LPI)	The percentage of the landscape comprised by the largest patch of the corresponding patch type
Aggregation Index (AI)	Measures the number of like adjacencies of corresponding class from 0 (no adjacencies) to 100
Simpson's diversity index (SIDI)	Simpson's diversity index is based on the proportional abundance of each land-use type
Interspersion and juxtaposition index (IJI)	Measure of evenness of patch adjacencies equals 100 for even and approaches 0 for uneven adjacencies
Landscape shape index (LSI)	A perimeter-to-area ratio that measures the overall geometric complexity of the landscape

## Declaration of Competing Interest

The authors declare that they have no known competing financial interests or personal relationships which have, or could be perceived to have, influenced the work reported in this article
